# Adherence to Vaccination Policy among Public Health Professionals: Results of a National Survey in Italy

**DOI:** 10.3390/vaccines8030379

**Published:** 2020-07-11

**Authors:** Maria Teresa Montagna, Osvalda De Giglio, Christian Napoli, Fabrizio Fasano, Giusy Diella, Rosalba Donnoli, Giuseppina Caggiano, Silvio Tafuri, Pier Luigi Lopalco, Antonella Agodi, GISIO-SItI Working Group

**Affiliations:** 1 Department of Biomedical Science and Human Oncology, University of Bari Aldo Moro, Piazza G. Cesare 11, 70124 Bari, Italy; mariateresa.montagna@uniba.it (M.T.M.); fabrizio.fasano1979@libero.it (F.F.); giusy.diella@uniba.it (G.D.); giuseppina.caggiano@uniba.it (G.C.); silvio.tafuri@uniba.it (S.T.); 2 Department of Medical Surgical Sciences and Translational Medicine, “Sapienza” University of Rome, Via di Grottarossa 1035/1039, 00189 Rome, Italy; christian.napoli@uniroma1.it; 3 Department of Prevention, ASL Bari, 70122 Bari, Italy; rosalba.donnoli@asl.bari.it; 4 Department of Translational Research and New Technologies in Medicine and Surgery, University of Pisa, Via Savi 10, 56126 Pisa, Italy; pierluigi.lopalco@unipi.it; 5 Coordinator of GISIO-SItI Working Group, Department of Medical and Surgical Sciences and Advanced Technologies “GF Ingrassia”, University of Catania, 95123 Catania, Italy; agodia@unict.it; 6 GISIO-SItI Working Group–Italian Study Group of Hospital Hygiene–Italian Society of Hygiene, Preventive Medicine and Public Health, Viale Cittá d’Europa, 74, 00144 Rome, Italy; martina.barchitta@unict.it

**Keywords:** public health professional, healthcare workers, knowledge, recommended vaccinations, attitude, vaccination coverage, machine learning

## Abstract

Starting from 2013, the number of unvaccinated people alarmingly increased in Italy; therefore, in 2017 a new Vaccine National Plan was approved. Healthcare workers (HCWs), especially public health professionals (PHPs, i.e., workers in in the sector of hygiene and preventive medicine), have an important role in informing and promoting vaccinations. In this context, the Italian Study Group of Hospital Hygiene of the Italian Society of Hygiene, Preventive Medicine and Public Health (GISIO-SItI) conducted a national survey to assess knowledge, attitude, and practices towards recommended vaccinations among PHPs. The survey was conducted during October 2019 with an anonymous questionnaire distributed to PHPs attending the 52° SItI National Congress. Overall, 57.1% of operators answered correctly to all seven recommended vaccinations, 12.8% reported to be vaccinated for all seven recommended vaccinations, while 30% were naturally immunized. A higher immunization coverage was reported for anti-hepatitis B (88.9%) and measles (86.1%), and 81.3% of the participants reported being offered the influenza vaccination during the 2018/2019 season. The majority of our sample indicated that hepatitis B (95%) and influenza (93.7%) were the recommended vaccines for HCWs, while less was known regarding varicella, pertussis, diphtheria, and tetanus boosters every 10 years. PHPs who were vaccinated (or who intended to be vaccinated) were more likely to recommend vaccinations to their patients and provided a reassuring example to those hesitant patients. Finally, this is the first study that identified good algorithms (using the techniques of machine learning as Random Forest and Deep Learning) to predict the knowledge of PHPs regarding recommended vaccinations with possible applications in other national and international contexts.

## 1. Introduction

Vaccines are considered one of the most effective and safest preventive measures at population level, followed by screening intervention [1,2,3,4]. Nevertheless, in Italy, starting from 2013, the number of under-vaccinated or unvaccinated people alarmingly increased because of currently well-known phenomenon of “vaccine hesitancy” defined by the World Health Organization Strategic Advisory Group of Experts (WHO SAGE) working group as the “delay of acceptance or refusal of vaccination despite availability of vaccination services” [2,5,6,7,8,9]; in this scenario, control and elimination strategies could fail [3,4,5,6,7,8,9,10].

Decreasing immunization coverage caused the onset of cases or outbreaks of vaccine-preventable diseases (VPDs), such as *Haemophilus influenzae* b invasive diseases, varicella, pneumococcal infections, measles, pertussis, influenza, and its corresponding complications [5,11].

In 2017, a new National Plan for Vaccine Prevention (NPVP) was approved in Italy [12], followed in the same year by Law 119/2017 [13], which declared 10 mandatory vaccinations for diphtheria, tetanus, hepatitis B, poliomyelitis, *Haemophilus influenza* type b, pertussis, measles, mumps, rubella, and varicella (MMRV). The NPVP led to important policy changes, including the introduction of new vaccine recommendations (offered actively and free of charge) and the addition of new target populations, such as adults at a higher risk of infection and the elderly. However, further efforts are necessary to reach other vulnerable groups, including healthcare workers (HCWs) [14]. In particular, the new NPVP recommends actively immunizing HCWs against hepatitis B, influenza, MMR, varicella, and acellular pertussis, and it encourages HCWs to receive diphtheria and tetanus booster doses every 10 years [12].

In order to increase adherence to vaccination recommendations, information campaigns and communication strategies should be implemented. In this context, HCWs, especially public health professionals (PHPs, i.e., working in the sector of hygiene and preventive medicine), have an important role in informing, advising and promoting vaccinations, contrasting vaccine safety fears, tackling vaccine hesitancy, and emphasizing the benefits and value of vaccines [15]. Although spread of infectious agents within healthcare settings represents a public health problem and one of the most important drivers of antimicrobial resistance, the risk of acquiring a VPDs in healthcare settings is not perceived to be particularly high [16]. Therefore, knowledge and practice concerning vaccines and vaccination strategies among PHPs are of fundamental importance to promote a culture of vaccination in the general population. HCWs are one of the most at-risk groups for acquiring infectious diseases and transmitting them to other hospital staff and patients, particularly to vulnerable groups [17]. Thus, there is a need to promote vaccination practice among HCWs to achieve control of vaccine-preventable diseases [14].

In Italy, several studies have been conducted on HCW vaccination coverage [18,19,20,21,22], but few studies have investigated the knowledge and risk perception of vaccinations [23,24,25,26], and to our best knowledge, none specifically addressed PHPs.

The Italian Society of Hygiene, Preventive Medicine and Public Health (SItI), a group of more than 2000 PHPs, engages in promoting and protecting citizens’ health through vaccination, among other practices. Therefore, the Italian Study Group of Hospital Hygiene of the SItI (GISIO-SItI) conducted a national survey to assess knowledge, attitudes, and practices towards recommended vaccinations among PHPs.

## 2. Materials and Methods

### 2.1. Study Design

The survey was conducted in October 2019 with an anonymous questionnaire distributed during the 52° SItI National Congress, to PHPs that were a part of the SItI. A sample of at least 968 enrolled individuals would have been required to investigate the selected variables in the PHPs population examined. The sample was calculated by a sample size calculator, based on the reference population of SItI members (*N* = 2030), and assuming a response proportion of 50% [23], a 99% confidence level and a 3% margin error.

The participants were enrolled on a voluntary basis after consent (as required by Italian privacy law) inferred by completion of the questionnaire, and they were not remunerated for their contribution. This questionnaire was accompanied by an information sheet that outlined the rationale for the study and explained that participation was voluntary, that the compilation took a few minutes, that they would have to deliver answers immediately and that full confidentiality and anonymity would be maintained. No personally identifiable information was collected in the questionnaire.

The investigation was performed in accordance with the World Medical Association’s Declaration of Helsinki and did not include any experiment involving human or biological human samples, nor research on identifiable human data. Therefore, the study protocol received ethical and scientific approval by Department of Biomedical Science and Human Oncology, University of Bari Aldo Moro (Bari, Italy) with the number 0407_2019.

### 2.2. The Questionnaire

The questionnaire consisted of multiple-choice questions divided into two sections. In the first part of the survey, there were seven questions about demographic and personal characteristics (i.e., age, sex, region), professional setting, and occupation. The second part of the survey consisted of 14 questions about PHPs’ knowledge (*N* = 2) and attitude (*N* = 9) regarding the recommended vaccinations for HCWs and their immunization practices (*N* = 3). Three questions pertaining to attitudes were measured on a seven-point Likert scale with options from “strongly disagree” to “strongly agree.”

Answering to all questions was compulsory. The questionnaire was previously tested in a pilot study on a sample of 54 PHPs at the University of Bari Aldo Moro. The reliability index was assessed using Cronbach’s alpha (internal consistency coefficient). The alpha values achieved were 0.975 and 0.972 for the pilot study and for the multicenter study, respectively. Data from the pilot were included in the final analysis.

### 2.3. Statistical Analyses

The information collected was entered into a database (Google forms) and analyzed using R version 3.6.3. (Bell Laboratories, formerly AT&T, now Lucent Technologies, Murray Hill, NJ, USA). A descriptive analysis was performed considering demographic and professional characteristics of participants and answers provided by the sample. Data regarding conditions and behaviors were reported as the number and percentage of respondents; these variables were compared between the two groups “agreement” and “disagreement” using the Chi-squared test. A *p*-value < 0.05 was regarded as statistically significant.

In order to evaluate the possible influence on the variable “knowledge of recommended vaccinations for HCWs” (dependent variable), the results of seven responses on recommended vaccinations were grouped in one scored variable. The value of this variable was represented by the sum of the correct answers for each operator, assigning one point for each right question. Subsequently, ranges of scores were identified as follows:

- From 0–3 correct answers (Low knowledge evaluation—Attribution Value = 1);
- From 4–6 correct answers (Medium knowledge evaluation—Attribution Value = 2);
- 7 correct answers (High knowledge evaluation—Attribution Value = 3).

Subsequently, the variables not related to knowledge of the recommended vaccinations were not considered (for example “If you are not vaccinated/immunized naturally for MMRV, are you going to get vaccinated in the coming months?”). The result was the selection of 19 independent variables. Descriptive responses were coded to numbers (ordinal coding) [27,28] in order to carry out a multivariate analysis to understand their influence on knowledge. The variables and identification codes are listed below:

- Sex of the respondents (1 = Male, 2 = Female);
- Age of the respondents;
- Length of practice;
- Professions of the HCWs (1 = Physicians, 2 = Biologists, 3 = Nursing Sciences, 4 = Healthcare Assistant, 5 = Prevention Technician, 6 = Other degree);
- Professional Setting (1 = Hospital, 2= Prevention Department, 3= District, 4 = Others);
- Operative Units/Service (1 = Medical management, 2 = Hospitalization facilities, 3 = Other direct services to patients, 4 = Epidemiology and Biostatistics, 5 = Public Hygiene and Health Service, 6 = Food and Nutrition Hygiene Service, 7 = District Management);
- Region of the HCWs (in order to tabulate Region, a number was assigned from 1–19);
- Are you vaccinated or naturally immunized against Hepatitis B? (1 = Vaccinated, 2 = Not vaccinated, 3 = Naturally immunized, 4 = I don’t know/I don’t remember);
- Are you vaccinated or naturally immunized against Measles? (1 = Vaccinated, 2 = Not vaccinated, 3 = Naturally immunized, 4 = I don’t know/I don’t remember);
- Are you vaccinated or naturally immunized against Mumps? (1 = Vaccinated, 2 = Not vaccinated, 3 = Naturally immunized, 4 = I don’t know/I don’t remember);
- Are you vaccinated or naturally immunized against Rubella? (1 = Vaccinated, 2 = Not vaccinated, 3 = Naturally immunized, 4 = I don’t know/I don’t remember);
- Are you vaccinated or naturally immunized against Varicella? (1 = Vaccinated, 2 = Not vaccinated, 3 = Naturally immunized, 4 = I don’t know/I don’t remember);
- Are you aware of the existence of a structured protocol for the vaccination of HCWs where you work? (0 = No, 1 = Yes);
- I have no opinion on vaccinations (rating from 0–7);
- I believe that the risk of contracting a preventable vaccine disease and transmitting it to a patient is low (rating from 0–7);
- I believe that vaccinations by health professionals are a prerequisite for working in the health sector and being suitable for the job (rating from 0–7);
- It is better to acquire natural immunity to diseases rather than through vaccinations (rating from 0 to 7);
- I fear the possibility of adverse events resulting from vaccinations (rating from 0–7);
- Do you think it is necessary to implement the knowledge of health professionals about vaccinations? (rating from 0–7).

Machine learning algorithms, in particular those of Random Forest [29] and Deep Learning [30,31], were tested, taking simultaneously into consideration the 19 independent variables, in order to predict the classification variable “correct responses to recommended vaccinations” (classification from 1–3).

The 1050 questionnaires collected were randomly mixed in order to test these algorithms. Then, all independent variables were normalized, to make the different units of measurement uniform and comparable, through the following formula [32]:

Xn = (Xnn − Min(X)) ÷ Max(X) − Min(X)(1)

where: Xn normalized: value of each variable at record *n*; Xnn not normalized: value of each variable at record *n*; Max(X): max value of each variable; Min (X): min value of each variable.

Normalization of variables returned values in the dataset codified between 0 and 1. The first part containing 70% of the data was used to “train” the system (training phase), the second part containing 30% of the data was subsequently used to evaluate the quality of the model (testing phase) [33].

In order to run Deep Learning models, Rectified Linear Unit (ReLU) was used as the activation function. The “softmax” function at the output layer was used to better classify variables [34,35] and to reduce the influence of extreme values or outliers on the data without removing them from the dataset. Data were transformed in a nonlinear manner using a sigmoid function. For each layer of the model, a bias neuron was provided to strengthen its effectiveness because it is a constant that helps the model to better adapt to the given data.

To assess the accuracy of the developed models, confusion matrices were used between the predictive values of the models and the real values of the part of the testing dataset. The accuracy of the model was calculated by comparing the number of data correctly classified by the model compared to the real data as numerator and the number of data of the whole dataset as denominator.

To evaluate the order of importance of the variables and related graphic processing, the permutation-based VI scores method [36] was used. For this method, in particular, the package “vip” version 0.1.3.9000 of the program R (Brandon Greenwell, Cincinnati, Ohio) was used [37].

Lastly, an analysis was conducted to test the variables influencing the immunization status according to sex, age, profession, length of practice, occupation setting, operating unit, region, and presence of existing structured protocol for vaccination at their work structures. For this analysis, a Poisson regression model was applied.

For the application of inferential statistics analysis, the Shapiro–Wilk test was applied; for the verification of normality, Wilcoxon Mann–Whitney, Kruskal–Wallis, Dunn post hoc, Fisher’s F, and Chi-squared tests were used. All statistical tests were two sided.

## 3. Results

Among the total of 2030 participants to National Congress SItI, 1050 PHPs agreed to participate and returned the survey for an overall response rate of 51.7%. The main demographic and professional characteristics of the study participants are displayed in Table 1. The mean age was 44.9 years (range 23–80), 58.6% were female and the majority (63.1%) were physicians. With regard to seniority, the mean value of length of practice was 17.2 years (range 1–49). Working setting and geographic area of origin are also reported in Table 1.

Table 2 reports the answers of PHPs about HCWs recommended vaccinations. The range went from 95% affirmative responses on hepatitis B to 29.6% on hepatitis A vaccination. Overall, 600 operators out of 1050 (57.1%) answered correctly to all seven recommended vaccinations.

Overall, 12.8% of PHPs reported being vaccinated for all seven recommended vaccinations, while 30% were naturally immunized. In particular, 2% were naturally immunized for Hepatitis B, 44.5% for Measles, 40.3% for Mumps, 37.9% for Rubella (39.2% of women), and 65.4% for Varicella. Table 3 shows the immunization history reported by PHPs regarding the recommended vaccinations excluding naturally immunized. A higher immunization coverage was reported for anti-hepatitis B (88.9%), followed by Measles (86.1%), Rubella (79.0%), and Varicella (76%). Moreover, 34.2% (39/114) of PHPs were not vaccinated for hepatitis B (or did not remember) and 36% (87/242) of those not vaccinated (or did not remember) for MMRV declared that they will get vaccinated in the next months.

Overall, 81.3% (854/1050) of the participants were recommended to get influenza vaccination during the 2018/2019 season. Recommendations were mostly made by the Occupational medicine services (26.6%; 227/854) or by other colleagues (24.1%; 206/854) mainly through communication by the Health Authority (50.4%; 430/854).

**Figure 1:**
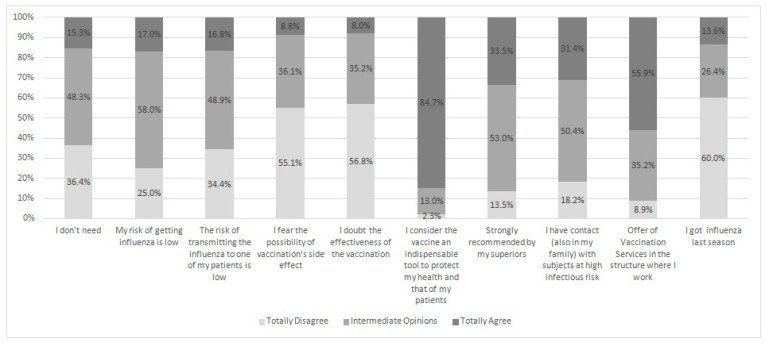
Attitudes of public health professionals (PHPs) (%) about refusing (from first to fifth answer) and adherence (from sixth to tenth answer) to anti-influenza vaccination.

Figure 1 reports PHPs’ attitude about anti-influenza vaccination. Between the agreement and disagreement on the first five reasons proposed in the questionnaire to refuse vaccination, the disagreements prevailed (χ^2^ = 40.6, *p* < 0.0001; χ^2^=6.7, *p* = 0.009; χ^2^ = 28.7, *p* < 0.0001; χ^2^ = 173.6, *p* < 0.0001; χ^2^ = 191.9, *p* < 0.0001, respectively in five reasons). Full agreement response to adherence to anti-influenza vaccination were more frequently reported (χ^2^ = 963.7, *p* < 0.0001; χ^2^ = 78.1, *p* < 0.0001; χ^2^ = 32.5, *p* < 0.0001; χ^2^ = 351.9, *p* < 0.0001, respectively, from the sixth to the ninth reason). The last reason showed the prevalence of disagreeing responses (χ^2^ = 323.2, *p* < 0.0001). In particular, 84.7% of enrolled PHPs considered anti-influenza vaccination an important tool to protect themselves and their patients.

A total of 65.5% (688/1050) of PHPs reported a structured protocol for vaccination at their workplace, and 60% (768/1050) of the sample recommended vaccinations to their patients, according to new NPVP, during medical practice.

**Figure 2:**
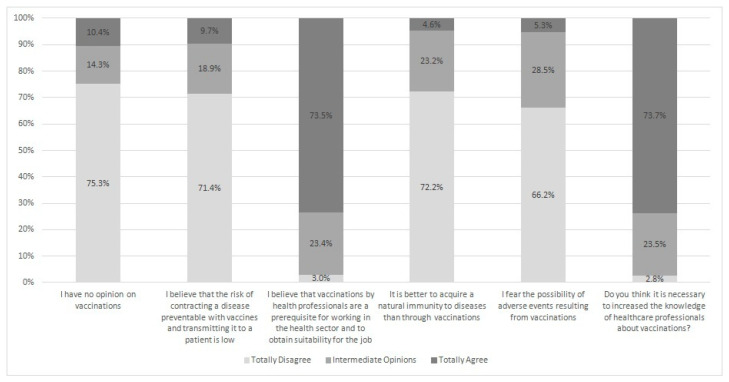
PHPs’ opinions (%) about vaccination practice.

Figure 2 reports PHPs’ opinions on vaccination practice. Most of them fully agreed that vaccinations for HCWs are an indispensable prerequisite to working in a healthcare environment (73.5%) and that their knowledge should be increased (73.7%).

Table 4 reports the inferential statistical analysis conducted to establish to what extent PHPs’ vaccination administration influenced the answer to the question “In your clinical practice do you recommend vaccinations to your patients?”.

PHPs that were vaccinated against hepatitis B suggested vaccination more often than those who were not vaccinated (*p* < 0.001). In the case of measles, mumps, rubella, and varicella, this difference was statistically significant both between vaccinated and unvaccinated and between naturally immunized and unvaccinated (*p* < 0.001).

Regarding predictive algorithms, Deep Learning had an accuracy on the testing part (303 questionnaires) from 50.3–65.6%. The Random Forest model (parameters ntree = 500, mtry = 10) had a better performance (73.6%) and was used to identify the order of importance of the 19 variables that had the greatest impact on the knowledge of HCWs regarding vaccinations. The most important variables influencing knowledge of recommended vaccinations were age (11.9%), length of practice (11.2%), region (10.9%), operating unit (6.9%), and profession (5%).

The model highlighted that the knowledge of recommended vaccinations is higher at a younger age, it drops to the lowest point around 45 years, and then it rises slightly. Similarly, the knowledge is higher at minimum and maximum duration of practice, while it tends to drop for PHPs with intermediate duration of practice (about 25 years). The Italian northern regions seem to have greater knowledge than the Italian central and southern regions. PHPs engaged in epidemiology and biostatistics and public hygiene and health service had greater knowledge of vaccinations. Finally, physicians were more aware of these vaccinations.

**Figure 3:**
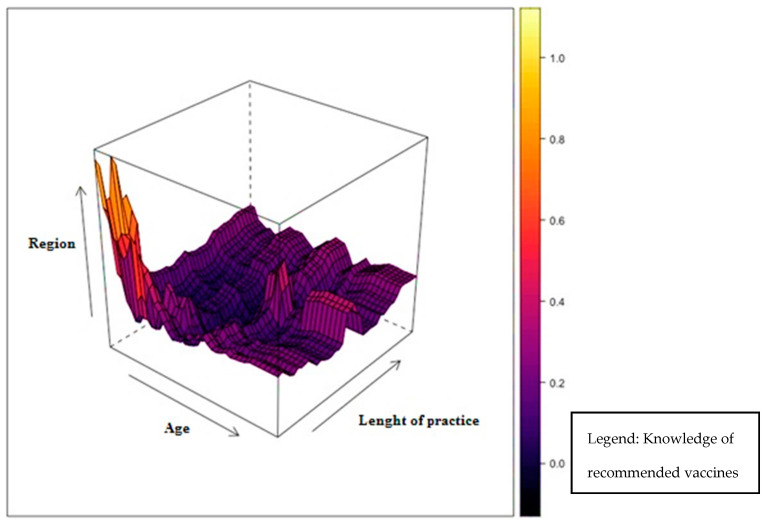
Model simulations according to Age, Length of practice, and Region variables versus knowledge of the recommended vaccinations.

Figure 3 shows a graph of the model simulations simultaneously considering the age, lengths of practice, and region variables with respect to knowledge of the recommended vaccinations. The key to reading the graph derives from the color palette. The greatest knowledge of vaccinations is shown in the clearest areas. The northern regions are represented by high numbers, while the southern ones are represented by low numbers. The figure identifies the prototype of the PHPs with a higher knowledge of recommended vaccinations in those with youngest age, less length of practice, and coming from northern Italy.

Inferential statistics tests on the above five variables were used in order to understand whether the simulation data corresponded to reality. No parametric tests were used because the distribution of the responses was not normal (Shapiro–Wilk test: W = 0.70, *p*-value < 0.0001).

Comparison between the respondents aged 23–38 years and the rest of the respondents confirmed that this age group was more aware of HCW recommended vaccinations (Wilcoxon rank sum test: W = 981734, *p*-value < 0.0001).

Comparison between the respondents for the classes of length of practice (0–10 years, 11–20 years, 21–30 years, 31–40 years, >40 years) revealed a significant difference (Kruskal–Wallis χ^2^ = 22.01, df = 4, *p*-value = 0.0001). In particular Dunn’s post hoc test confirmed that 0–10 years of practice was more aware of HCW recommended vaccinations compared to 11–20 years (Dunn test = 3.02, *p*-value = 0.001), 21–30 years (Dunn test = 3.80, *p*-value = 0.0001), and 30–40 years (Dunn test = 3.45, *p*-value = 0.0003).

Comparison between geographical areas (North, Central, and South Italy) revealed a significant difference (Kruskal–Wallis χ^2^ = 13.12, df = 2, *p*-value = 0.001). In particular, through Dunn’s post hoc test, it was noted that the Northern Italian regions knew more about vaccinations than Central Italian Regions (Dunn test = 2.60, *p*-value = 0.046) and Southern Italian Regions (Dunn test = 3.44, *p*-value = 0.0003).

For the Operating Units (OUs), the Kruskal–Wallis test (χ^2^ = 56.75, df = 6, *p*-value < 0.0001) revealed statistically significant differences. In particular, with Dunn’s post hoc test, the public hygiene and health service OUs were found to have greater knowledge of the recommended vaccinations compared to all other OUs (*p*-values < 0.05).

Finally, regarding professions, the Kruskal–Wallis test confirmed the statistical significance of the results (χ^2^ = 59.34, df = 5, *p*-value < 0.0001). In particular, Dunn’s test highlighted how physicians were more aware of the recommended vaccinations than biologists (*p*-value < 0.0001) and nurses (*p*-value < 0.0001).

The Poisson Regression Model led to similar results of Random Forest Model. The younger age (β = −0.019, OR = 0.98, 95% CI = 0.97–0.99, p = 0.0006) and the less length of practice seems to influence more aware of HCW recommended vaccinations (−β = −0.023, OR = 0.97, 95% CI = 0.96–0.98, *p*-value < 0.0001). Regarding Region, Northern Italy Regions are more aware of HCWs’ recommended vaccinations (−β = −0.008, OR = 0.99, 95% CI = 0.98–0.99, *p*-value = 0.02). Moreover, other variables influencing positively aware of HCW recommended vaccinations were the existence of a structured protocol for the vaccination of HCWs in the work structures (β = 0.18, OR = 0.86, 95% CI = 0.92–0.80, *p*-value < 0.0001), having less fear of adverse events resulting from vaccinations (β = −0.04, OR = 0.95, 95% CI = 0.98–0.93, *p*-value < 0.0001), and having done the vaccination against Hepatitis B (β = −0.14, OR = 0.86, 95% CI = 0.92–0.80, *p*-value < 0.0001).

The last analysis was conducted to verify which variables influenced each type of recommended vaccination among PHPs, and results are reported below (Poisson Regression Model):

- Anti-hepatitis B vaccination was associated with younger age (β = −0.003, OR = 0.996–95% CI = 0.997–0.994, *p* < 0.0001), profession (β = −0.047, OR = 0.95–95% CI = 0.97–0.94, *p* < 0.0001), and presence of existing structured vaccination protocol in their work structures (β = 0.0846, OR = 1.08–95% CI = 1.14–1.04, *p* < 0.0001);
- Anti-measles vaccination was associated with male sex (β = −0.0801, OR = 1.08–95% CI = 1.17–1.004, *p* = 0.04) and older age (β = 0.013, OR = 1.013–95% CI = 1.016–1.010, *p* < 0.0001);
- Anti-mumps vaccination was associated with older age (β = 0.0084, OR = 1.008–95% CI = 1.011–1.005, *p* < 0.0001), profession (β = −0.048, OR = 0.95–95% CI = 0.98–0.92, *p* = 0.003), and operational units (β = 0.039, OR = 1.04–95% CI = 1.06–1.02, *p* < 0.0001);
- Anti-rubella vaccination was associated with female sex (β = 0.1483, OR = 1.07–95% CI = 1.26–1.16, *p* = 0.0004), older age (β = 0.009, OR = 1.009–95% CI = 1.012–1.006, *p* < 0.0001), profession (β = −0.0364, OR = 0.96–95% CI = 0.99–0.93, *p* = 0.02), operational units (β = 0.0299, OR = 1.03–95% CI = 1.05–1.01, *p* = 0.005), and presence of existing structured vaccination protocol in their work structures (β = 0.0875, OR = 1.09–95% CI = 1.19–1.00, *p*-value = 0.05);
- Anti-varicella vaccination was associated with female sex (β = 0.0967, OR = 1.10–95% CI = 1.19–1.02, *p* = 0.02) and profession (β = −0.0340, OR = 0.97–95% CI = 0.99–0.94, *p*-value = 0.02);
- Anti-diphtheria–tetanus–pertussis vaccination was associated with profession (β = −0.0403, OR = 0.96–95% CI = 0.98–0.94, *p* = 0.0001), region (β = −0.0115, OR = 0.99–95% = CI 0.99–0.98, *p* < 0.0001), and presence of existing structured vaccination protocol in their work structures (β = 0.1455, OR = 1.16–95% CI = 1.23–1.09, *p*-value < 0.0001);
- Anti-influenza vaccination was associated with male sex (β = −0.0598, OR = 0.94–95% CI = 1.00–0.89, *p* = 0.04), older age (β = 0.0040, OR = 1.004–95% CI = 1.006–1.002, *p* = 0.0004), profession (β = −0.0436, OR = 0.96–95% CI 0.98–0.94, *p* < 0.0001), and presence of existing structured vaccination protocol in their work structures (β = 0.2130, OR = 1.24–95% CI = 1.31–1.17, *p*-value < 0.0001).

## 4. Discussion

Several surveys have been carried out worldwide on the knowledge, attitudes, and practices of HCWs in relation to vaccinations [23,38,39], but very few have specifically studied PHPs [22].

According to our experience, referring to PHPs, 57.1% of the study sample was aware of all recommended vaccinations for HCWs (with a higher degree of knowledge in northern regions than in southern regions), unlike some other Italian studies that included all HCWs and reported lower values [23,24].

Younger PHPs and with fewer years of practice had more knowledge about recommended vaccinations; this may be due to the fact that less time had passed since obtaining their degree. Furthermore, consistent with other studies [24,40], physicians showed significantly higher levels of knowledge compared to nurses, partially due to their education being focused mostly on patient care and with inadequate time given to vaccinations. Therefore, nursing education curricula should be evaluated to ensure that adequate time is given to vaccine training.

Overall, the majority of our sample indicated that hepatitis B and influenza were recommended vaccines for HCWs, as reported in previous national investigations [23,24], while a lower level of knowledge was reported for anti-MMRV, especially for varicella, and for the need of diphtheria and tetanus boosters every 10 years.

Inadequate knowledge among PHPs may result in the transfer of false information to patients and the public. Therefore, it is important to continue implementing educational campaigns for PHPs, and in general for HCWs, on vaccination because they have been consistently identified as the most influential and trusted source of information on vaccine-preventable infectious disease [24,41].

In our study, only 12.8% of PHPs received all seven recommended vaccinations, and immunization status revealed inadequate vaccine coverage, i.e., below 95% for all recommended vaccinations examined. This result is not comforting, since, despite the significant impact of the media, PHPs have been identified as the most important source of information on vaccination for the general public; therefore, the willingness of health professionals to recommend immunization is crucial [42]. PHPs among all HCWs should promote immunization in different health care settings and develop effective communication toolkits and educational materials [24,43]. Indeed, in our study, only 60% of PHPs recommended vaccinations to their patients during medical practice.

In line with previous studies [23,24,26], high rates of coverage were self-reported, even if still sub-optimal for hepatitis B and measles, whereas they were lower for rubella, varicella, mumps, influenza, and diphtheria and tetanus boosters every 10 years.

Consistent with other studies [20], younger PHPs had higher reported vaccination coverage for hepatitis B, which might be explained by the fact that, recently, more attention has been paid to parenterally transmitted diseases among HCWs.

Regarding measles and rubella, a reported high vaccination coverage was found in our survey, thanks to the National Plan of Measles and Congenital Rubella Elimination (PNEMoRC) 2010–2015, which strongly recommended vaccination of susceptible adults at risk of contracting and transmitting disease, such as health professionals [44]. In fact, our survey showed that most PHPs contracted measles (44.5%) and varicella (65.8%) during their lifetimes because both diseases were endemic in some Italian regions [18]. Occupationally acquired mumps, measles, varicella, rubella, and pertussis can be associated not only with prolonged morbidity in HCWs and patients but also with the occurrence of severe complications [45].

In our study, women reported significantly higher vaccination coverage for rubella compared to men. This is consistent with the fact that, in Italy, rubella vaccination has been offered free of charge to women in adolescence and childbearing age since the seventies. In our sample, 39.2% (241/615) of women were naturally immunized towards rubella disease.

Concerning influenza, it has been demonstrated that about 25% of HCWs contract the virus every year; thus, they can transmit the infection to patients, especially during the asymptomatic period [46]. Knowledge and practice towards influenza vaccination among HCWs is important not only to reduce the burden of morbidity and mortality [47], but also to decrease the related remarkable costs for the National Health Service related to the care of ill people and, especially, consequent decreased productivity due to absence from work [48].

Compared to previous studies conducted in some European countries including Italy and targeted to HCWs [19,23,24,49,50], we found a higher percentage of vaccination coverage (66.5%) for influenza probably because our study included PHPs. Nevertheless, PHPs’ reported vaccination coverage for seasonal influenza in 2018/2019 was lower than the target of 75% to be achieved by 2015 put forth by the EU Council recommendation of December 2009 [51]. According to other studies, possible reasons to decline influenza vaccination may be its ineffectiveness [52,53] and fear of side effects [44].

Our findings revealed that older workers had a higher likelihood to have been vaccinated for influenza, which is consistent with findings from other authors [19,20], probably because older HCWs are also recommended due to age. In accordance with other studies [23], 84.7% of PHPs considered influenza vaccination useful to protect their own health and their patients; therefore, effective risk communication is indispensable to promote adequate adherence to vaccination. In fact, despite the various education initiatives promoted by SItI, in our study, PHPs reported that it is necessary to further increase operators’ knowledge about vaccinations (73.7%). Another study [20] showed that counseling and influenza vaccination proposals came mainly from Occupational health physicians (OHPs) (26.6%) and other colleagues (24.1%), especially through communication by the Health Authority (50.4%). OHPs should record reasons for vaccination refusal, since the use of a signed informed declination statement for those refusing vaccination has been recommended as a possible tool to stimulate HCW vaccination rates [54].

Our study showed that most PHPs believe vaccinations are a prerequisite for eligibility to work in the healthcare sector (73.5%), and most reported the existence of a structured protocol for HCWs vaccination in their workplace (65.5%), which was associated with being vaccinated for hepatitis B, rubella, diphtheria–tetanus–pertussis, and influenza.

Fortunately, only a minority of PHPs reported several reasons for not being vaccinated: they did not consider themselves at risk for VPDs, and they had uncertainty regarding vaccine safety, such as in other studies regarding HCWs [46]. These barriers and misbeliefs among PHPs need to be addressed because there is a risk that PHPs who are hesitant about vaccines may spread concerns to the public and recommend vaccines less frequently. On the other hand, in accordance with several studies [55], PHPs in our study who were vaccinated (or who intended to be vaccinated) were more likely to recommend vaccination to their patients, which provides a reassuring example to those patients who have concerns. In particular, PHPs that had anti-hepatitis B vaccinations were found to suggest vaccination more often than PHPs who were not vaccinated. This finding may be related to the fact that they did not believe in severe adverse effects from vaccines and had not doubts about the benefits of vaccination. In the case of measles, mumps, rubella, and varicella, this difference was statistically significant both between the vaccinated and unvaccinated and also between the naturally immunized and unvaccinated. It is probable that the experience and higher level of knowledge of such diseases leads to greater patient safety concerns [55].

On the other hand, the current Covid-19 pandemic and the great expectance for a vaccine [56] could represent an opportunity to fight the propensity of vaccine resistance among Italian HCWs and, in particular, PHPs heavily hit by the virus.

The authors are aware of some potential limitations of this study. Firstly, the cross-sectional nature limits inferences on the directional causality of the associations observed in this study. Secondly, no information was given about no-responders (e.g., type of degree, age groups) since they did not give their consent to participate to the survey. Therefore, the conclusion of the study might not be generalizable to all Italian PHPs. Thirdly, the information about vaccination status was self-reported rather than record-review. This might be prone to recall, declaration, or desirability biases; therefore, an over- or underestimation of coverage could occur. However, the fact that the survey maintained full confidentiality and anonymity may suggest that the responses were likely to be authentic, with minimal social desirability bias.

Despite these potential biases, the wide response to the questionnaire (over 50% of the population) reduces the risk of the non-representativeness of the sample compared to the entire population. Finally, to our knowledge, this is the first study to identify good algorithms that are capable of predicting the knowledge of PHPs regarding recommended vaccinations, with possible applications in other national and international contexts. However, the algorithms can be improved with a higher degree of accuracy through more numerically conspicuous “training data” and consideration of other independent variables (sources of information about immunizations or data concerning socio-demographic characteristics). These considerations may be the basis for further research.

## 5. Conclusions

Our study provides useful insights for learned societies, such as SItI, which are in charge of supporting the implementation of evidence-based national-level vaccination policies and improving the knowledge of vaccination among PHPs. PHPs could promote immunization by removing prejudices and including educational and implementation strategies to improve vaccine adhesion and confidence among people. Finally, the implementation of algorithms could represent an innovative tool to better target educational campaigns.

## Figures and Tables

**Table 1:** Demographic and professional characteristics of Italian public health professionals (PHPs).

Characteristics	No. (%)	Sub-Category	No. (%)
Total	1050	-	-
**Sex**		-	-
Female	615 (58.6)	-	-
Male	435 (41.4)	-	-
**Age, years**	**N (%)**	-	-
18–24	8 (0.8)	-	-
25–34	293 (27.9)	-	-
35–44	197 (18.8)	-	-
45–54	231 (22)	-	-
55–64	272 (25.9)	-	-
>65	49 (4.6)	-	-
**Length of practice, years**	**N (%)**	-	-
0–10	414 (39.4)	-	-
11–20	178 (17.0)	-	-
21–30	262 (25.0)	-	-
31–40	174 (16.6)	-	-
>40	22 (2.0)	-	-
**Profession**	**N (%)**	-	-
Physicians	663 (63.1)	-	-
Biologists	153 (14.6)	-	-
Nurses	100 (9.5)	-	-
Healthcare Assistant	68 (6.5)	-	-
Prevention Technician	23 (2.2)	-	-
Others	43 (4.1)	-	-
**Workplace**	**N (%)**	**Operating Unit**	**No. (%)**
Hospital	454 (43.2)	Medical management	134 (12.8)
-	-	Hospital facilities	126 (12)
-	-	Other services to patients	109 (10.4)
-	-	Epidemiology and Biostatistics	85 (8.1)
Prevention Department	387 (36.8)	Public health services	253 (24.1)
-	-	Other services to patients	59 (5.6)
-	-	Food and nutrition hygiene service	41 (3.9)
-	-	Epidemiology and Biostatistics	34 (3.2)
Socio-sanitary District	48 (4.6)	District management	25 (2.4)
-	-	Other services to patients	23 (2.2)
Others	161 (15.3)	-	161 (15.3)
**Geographical distribution of participants in Italy**	**N (%)**	-	-
Northern	371 (35.3)	-	-
Central	261 (24.8)	-	-
Southern	418 (39.8)	-	-

**Table 2:** Correct responses of 1050 PHPs regarding the recommended vaccinations for healthcare workers (HCWs).

Recommended Vaccination	Yes (%)	No (%)
Influenza	984 (93.7)	66 (6.3)
Hepatitis B	997 (95.0)	53 (5.0)
Hepatitis A	311 (29.6)	739 (70.4)
Measles-Mumps-Rubella	916 (87.2)	134 (12.8)
Varicella	792 (75.4)	258 (24.6)
Pertussis	811 (77.2)	239 (22.7)
Tetanus	878 (83.6)	172 (16.4)
Diphtheria	795 (75.7)	255 (24.3)

**Table 3:** Immunization coverage of 1050 PHPs regarding recommended vaccinations for healthcare workers (HCWs).

Diseases	Vaccinated No. (%)	Not Vaccinated No. (%)	I Don’t Know/I Don’t Remember No. (%)
Hepatitis B	915 (88.9)	84 (8.2)	30 (2.9)
Measles	502 (86.1)	38 (6.5)	43 (4.1)
Mumps	473 (75.4)	82 (13.1)	72 (6.9)
Rubella	515 (79.0)	65 (10.0)	72 (6.9)
Varicella	276 (76.0)	44 (12.1)	43 (4.1)
Diphtheria-Tetanus-Pertussis	703 (67.0)	347 (33.0)	0
Influenza	698 (66.5)	352 (33.5)	0

**Table 4:** Comparison among PHPs recommending vaccinations according to immunization coverage (statistically significant differences in bold).

Immunization Coverage	Hepatitis B	Measles	Mumps	Rubella	Varicella
Vaccinated vs. Naturally immunized	(703/915–76.8%) vs. (14/21–66.7%) Fisher f–*p* = 0.32	(379/501–75.7%) vs. (20/39–51.3%) χ^2^ = 0.02 *p* = 0.90	(355/473–75.1%) vs. (339/423–80.1%) Fisher f–*p* = 0.22	(382/514–74.3%) vs. (522/687–79.9%) Fisher f–*p* = 0.07	(208/276–75.4%) vs. (522/687–76.0%) Fisher f–*p* = 1
Vaccinated vs. Not Vaccinated	(703/915–76.8%) vs. (40/84–47.6%) **χ^2^ = 12.58 *p* = 0.0004**	(379/501–75.7%) vs. (20/39–51.3%) **Fisher f–*p* = 0.0005**	(355/473–75.1%) vs. (48/83–57.8%) **χ^2^ = 12.4 *p* = 0.0004**	(382/514–74.3%) vs. (19/44–43.2%) **χ^2^ = 10.07 *p* = 0.001**	(208/276–75.4%) vs. (19/44–43.2%) **Fisher f–*p* < 0.0001**
Not Vaccinated vs. Naturally immunized	(40/84–47.6%) vs. 14/21–66.7%) **Fisher f–*p* = 0.67**	(20/39–51.3%) vs. (355/467–76.0%) **Fisher f–*p* = 0.001**	(48/83–57.8%) vs. (339/423–80.1%) **Fisher f–*p* = 0.0001**	(19/44–43.2%) vs. (522/687–79.9%) **Fisher f–*p* = 0.0001**	(19/44–43.2%) vs. (522/687–76.0%) **χ^2^ = 29.62 *p* < 0.0001**
